# A Literature Review: The Mechanisms and Treatment of Neuropathic Pain—A Brief Discussion

**DOI:** 10.3390/biomedicines12010204

**Published:** 2024-01-17

**Authors:** Renira Rugnath, Casey Orzechowicz, Clayton Newell, Veronica Carullo, Anesh Rugnath

**Affiliations:** Department and Anesthesiology, University of Mississippi Medical Center, Jackson, MS 39216, USA; rrugnath@umc.edu (R.R.);

**Keywords:** neuropathic pain, neuralgia, radiculopathy, sensitization, peripheral nervous system, pediatric pain, genetic pain, gabapentinoids, tricyclic antidepressants, capsaicin, spinal cord stimulators, neurostimulation, interventional pain, compression

## Abstract

Classically, neuropathic pain is described as a pain caused by a lesion or disease of the somatosensory system. However, one must note that the presence of somatosensory pathology alone does not guarantee a progression to neuropathic pain. This is due, in part, to the fact that neuropathic pain is a notoriously complex disease process, involving sensitization of both the central and peripheral nervous systems. Its causes are also numerous and varied, including trauma, the compression of a nerve, autoimmune disorders, diabetes, and infections. Due to the various manifestations, causes, and symptoms of neuropathic pain, the treatment of this disease process has proved challenging for generations of physicians. This section aims to elaborate on newly proposed mechanisms for pharmacological and targeted therapies, such as neurostimulation, which aim to reduce the negative somatosensory effects of neuropathic pain.

## 1. Introduction

**Figure 1:**
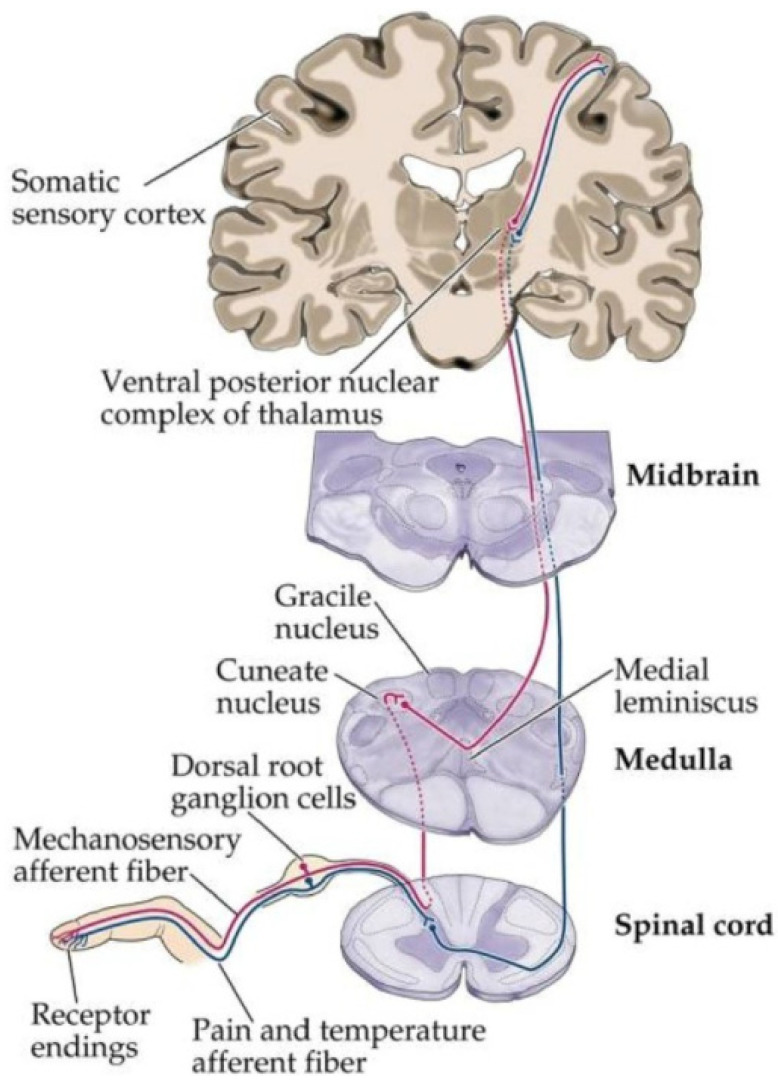
Possible sites of neuropathic pain lesions along the spinothalamic tract, including at the receptor level (receptor endings), peripheral nerve (afferent fibers), or spinal cord level or centrally within the brain (somatic sensory cortex) and brainstem (midbrain and medulla). Obtained from Reference [6] through an open-access archive.

The International Association for the Study of Pain (IASP) defines neuropathic pain as any uncomfortable sensation caused by a lesion or disease of the somatosensory system [1,2]. The hallmark signs and symptoms of neuropathic pain are a coupling of sensory disturbance or loss and associated pain with or without hypersensitivity at the site of pain [3,4]. Despite neuropathic pain recently coming into the public eye with curiosities such as phantom limb syndrome, it has been documented as far back as the American Civil War and continues to evolve in definition. Neuropathic pain, by definition, has been difficult to conglomerate and even diagnose due to the various presentations causes, symptoms, and underlying mechanisms. Throughout history, many proposed theories of pain have been offered to explain the various mechanisms via which neuropathic pain arises. One of the most popular theories, the Gate Control Theory, was first proposed in 1965 by Ronald Melzack and Patrick Wall [5]. This theory, though overly simplified, states that pain modulation can be either augmented or attenuated at the level of the spinal cord. More specifically, pain modulation occurs at the dorsal horn via regulatory pathways that primarily serve to provide appropriate negative feedback against signaling from the spinothalamic tract, which serves to transmit the sensation of pain and temperature. Despite uncertain pathogenesis, most researchers believe that neuropathic pain can originate at the receptor level, peripheral nerve, or spinal cord level or centrally within the brain and brainstem, as shown in Figure 1 [6]. Due to the vast scope of this topic, this discussion will encompass commonly proposed mechanisms, the pathophysiology of neuropathic pain states, and the current evidence-based recommendations for treatment.

## 2. Pathophysiology of Neuropathic Pain States

Neuropathic pain covers a broad spectrum of symptoms and presentations. Its descriptive adjectives have included burning, pins and needles, tingling, pressure, stabbing, squeezing, electric shock, or pain with touch, pressure, or temperature change. It can be further divided into categories of evoked, spontaneous, and referred pain.

### 2.1. Evoked Pain

**Figure 2:**
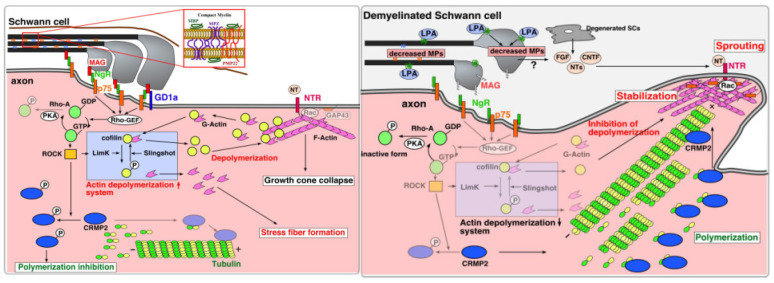
Schematic of fiber demyelination. Obtained from Reference [8] through an open-access archive.

Evoked pain, as the name suggests, is pain that requires a stimulus to elicit a pathologic pain response. Descriptors of evoked pain include the spread to nearby regions of uninjured tissue, hypersensitivity, allodynia, temperature change causing hot/cold allodynia, and touch hypersensitivity [5]. Intact and uninjured A-beta fibers in adjacent areas can become involved in the noxious pathways, with some studies suggesting that this may be in part due to A-beta-fiber sprouting, decreased C-fiber thresholds, and even alterations in ion signaling pathways. Some studies have even indicated that upwards of 50% of people with neuropathic pain experience some variation in evoked pain response, commonly brush-evoked allodynia and pressure-evoked pain [7]. A schematic from The Journal of Molecular Pain by Ueda et al., detailing the plasticity involved in neuropathic pain, is shown in Figure 2 [8].

### 2.2. Spontaneous Pain

Spontaneous pain is produced in areas of irritable and excitable somatosensory pathways, which can fire at random or in response to a temporal summation of varying physiological mechanisms. This slow-growing temporal summation and irritability partially account for the after-sensation and hyperpathic response seen after minor noxious stimuli [5]. Due to the contrasting nature of the initial presenting symptoms, physicians and researchers have tried to make sense of the variable expressions of pain. One such theory, the Label Line Theory, hypothesizes that the type of pain experienced depends on the type of sensory nerve fibers involved at the site of the initial injury [5,9,10]. Some studies suggest that Aβ/Aδ fiber damage will elicit sensations such as painful tingling, pricking, and throbbing [11]. In contrast, damage to unmyelinated C-fibers may lead to the constant burning sensation often experienced by patients suffering from neuropathic pain. Some studies suggest that the pain generators for some neuropathic conditions stem from areas such as the dorsal horn, dorsal root ganglion (DRG), or even the thalamus [5,12,13,14,15].

### 2.3. Referred Pain

Referred pain is pain that may be felt at an area other than the site of noxious stimuli. It has been linked to higher cortical sensory structures that will reorganize in the setting of trauma, leading to input from afferent nerves damaged by the injury [5,16]. Another proposed mechanism is nerve sprouting, causing a form of “cross-talk”, which could impact secondary-order neurons or even form connections that could invariably lead to disinhibition at the level of the descending modulatory pathways.

### 2.4. Central Pain

For this discussion, all central pain syndromes are grouped based upon the anatomic locations of injury. The most commonly seen central pain syndromes consist of post-stroke pain—which is most commonly seen in the thalamus—spinal cord injuries at and below the level of injury, and neurodegenerative disorders, such as multiple sclerosis (MS) and some subsets of amyotrophic lateral sclerosis (ALS). Loss of pain and temperature sensations is the hallmark symptom of all central pain syndromes generated via spinothalamic dysfunction [17,18]. Sensory hypersensitivity is also a primary marker for central pain but usually occurs after an acute spinal cord injury [19,20]. In both cases, the thought is that the primary mechanism of central pain occurrence results from dynamic changes within the pain regulatory system, as well as a global central sensitization leading to hypersensitivity and exaggerated pain responses [21]. This combined presentation of “negative” symptoms from pain and temperature loss and “positive” symptoms from sensory hypersensitivity is characteristic of central pain syndromes, with both the hyposensation and hypersensitivity of a localized area commonly experienced in a single patient, which suggests a two-fold etiology of the deafferentation of the ascending pathways in the spinothalamic tract and resultant neuronal hyperexcitability [18].

### 2.5. Peripheral Neuropathic Pain

#### 2.5.1. Post-Herpetic Neuralgia

Post-Herpetic Neuralgia (PHN) is a type of peripheral neuropathy (PN) that presents three months after a herpes zoster outbreak, and it is frequently described as a dermatomal burning, stabbing, shooting, or electric shock-like pain. Most researchers believe that the herpes zoster virus travels along the cranial nerve or spinal DRG nerve axons, which cause the inflammation and necrosis of the associated nerve [22,23,24], which supports the localized dermatomal pattern typically seen in herpes zoster. Estimates show that anywhere from 5 to 20% of people who experience a herpes zoster outbreak will develop chronic or relapsing episodes of PHN [5,25]. Some evidence shows that the pain associated with PHN is linked to altered membrane ion channels, specifically Na_v_1.6 and Na_v_1.7—which are known targets of many commonly used local anesthetics, demonstrating their efficacy in treating PHN [5,26].

#### 2.5.2. Trigeminal Neuralgia

Trigeminal Neuralgia (TN), also known as Tic douloureux, is commonly characterized by its spontaneous, electric shock-like pain that presents with a sudden onset and sudden discontinuance, typically lasting anywhere from seconds to minutes [27,28,29]. Mechanical stimuli such as touch, chewing, brushing teeth, or facial movements can incite a pain response; the location and stimuli vary depending on the trigeminal nerve distribution affected [27,30]. The classical mechanism via which TN occurs is considered by most researchers to result from the vascular compromise of the trigeminal nerve near the brainstem in the territories of the nerve root entry zone, the cistern segment, or the pontine segments [5,30,31]. Some researchers hypothesize that cross-sensitivity or “cross-talk” between A sensory fibers and C-nociceptive leads to the hypersensitivity and depolarization of the associated pain fibers.

#### 2.5.3. Painful Radiculopathy

Patients typically describe painful radiculopathy as a burning, squeezing, or pricking sensation, as well as possible hypersensitivity with associated sensory loss. Pain often presents after evoked stimuli, such as mechanical movement, touch, or pressure. Painful radiculopathy results from degenerative disk disease causing the compression of the DRG or disc herniation, causing direct spinal cord compression [5,27,32]. Some studies have even suggested that the inflammatory degradation products released from the nucleus pulposus can lead to nerve root inflammation and further contribute to the mechanical compression of the dorsal root.

#### 2.5.4. Painful Polyneuropathies

Painful polyneuropathy typically presents as an insidious onset of paresthesia and numbness, which may gradually progress to pain. This pain is commonly associated with the classic “stocking-glove” pattern. Polyneuropathies encompass the broadest range of diagnoses and diseases. The most notable of these are a result of diabetes, channelopathies, HIV, immune/autoimmune, and other genetic/biochemical imbalances that can lead to peripheral nerve injury [5,27,33,34]. The pathophysiology varies in mechanism depending on the underlying condition. Channelopathies such as the genetic Na_v_1.7 channel dysfunction occur in erythromelalgia, pathologic demyelination, or chronic vascular compromise, causing oxidative stress on the axons themselves [5]. This class encompasses a wide range of disorders with multiple possible mechanisms for developing neuropathic pain. Some research suggests that members of this class of neuropathic pain syndromes share a common feature, with spontaneous activity and hypersensitivity in occurring nociceptive C-fibers, along with associated central sensitization mechanisms and their neurophysiological changes.

#### 2.5.5. Peripheral Nerve Injury

Peripheral nerve injury typically results from a direct insult to a nerve or bundle of nerve cells, whether post-surgical or trauma induced. One such example is phantom limb syndrome in the setting of amputation. Several proposed theories suggest possible mechanisms for the development of pain. One of these theories is that neuronal sprouts may cause the sensitization of unaffected nerves, which some postulate is the reason for expanded areas of pain outside the injured zone. These hyperexcitable nerves can then cause central desensitization, as well as neuronal reorganization. Most researchers believe inflammation and immunological responses at the injury site can lead to hypersensitivity and altered membrane potentials [5,30].

## 3. Treatment

Though research continues in the field of neuropathic pain, the options for treatment have not significantly advanced in recent years. We will briefly discuss the current evidence-based recommendations for pharmacological and interventional options. There has been some variation in establishing standard treatment practices regarding first-, second-, and third-line treatments. However, the authors have found, through a thorough literature review, that the following options seem to be founded in evidence.

1. Pharmacologic interventions
  A First-line treatments
    - Gabapentinoids
      - Initially marketed as antiepileptic drugs, gabapentinoids, such as gabapentin and its precursor pregabalin, are somewhat effective in managing neuropathic pain symptoms. Their primary mechanism of action is activity at the α_2_δ subunit of voltage-gated calcium channels [30], which researchers believe prevent the release of neuro-excitatory molecules, thereby decreasing central sensitization. Some of the literature shows that gabapentinoids significantly relieved pain scores in diabetic peripheral neuropathy and postherpetic neuralgia [35,36]. The most commonly seen side effects of this class of medication are somnolence, weight gain, dizziness, ataxia, and fatigue. Contraindications primarily include dose-related issues seen in individuals with kidney disease and chronic alcohol use/abuse.
    - Tricyclic antidepressants
      - Tricyclic antidepressants (TCAs) were initially marketed as a class of antidepressants in the late 1950s. However, they were first seen to be helpful in conditions such as diabetic neuropathy as early as the 1960s. This class of drug’s primary mechanism of action is the prevention of the reuptake of monoamines. However, it has many other activities in the body, including sodium channel blocking and anticholinergic and anti-histaminergic activities. Therefore, some even propose that there could be other sites of action that have not yet been described [30]. The proposed site of action for pain treatment supposedly works through the activation of descending modulatory pain pathways, both in the spine and higher cortical areas. As this class of drugs has a diverse pharmacologic profile with its many sites of action, its use in the general population has been met with some hesitancy due to the vast side effect profile. For example, anticholinergic side effects include dry eyes, sedation, and urinary retention. Anti-histaminergic side effects include weight gain and somnolence. Sodium channel blockade can block cardiac fast sodium channels, leading to a widening of the QRS complex, arrhythmias, and even cardiac arrest. Contraindications for this drug are typically a result of an unwanted side effects. For this reason, one should take extra care when prescribing these medications to the geriatric population, those with heart conditions, especially conduction abnormalities, and glaucoma, as this can cause an acute exacerbation.
    - Serotonin-Norepinephrine reuptake inhibitors
      - Initially marked as a class of antidepressants, serotonin-norepinephrine reuptake inhibitors (SNRIs) have proven to have a modest side effect profile while also being effective in the treatment of neuropathic pain. By inhibiting the presynaptic reuptake of serotonin and norepinephrine, SNRIs act on the descending aminergic pathways in the spinal and supraspinal regions, decreasing central sensitization. The most common side effects are nausea, vomiting, headache, and insomnia [5,27]. Mood disturbances, serotonin syndrome, and an increased risk of suicide are among the most severe reactions. Some of the relative contraindications affect those who are on MOA inhibitors, the elderly, and those with severe liver/kidney issues.
  B Second-line treatments
    - Lidocaine patch
      - Lidocaine, first discovered in the 1940s, is an amide local anesthetic that exerts its primary mechanism of action on voltage-gated sodium channel blockade. The most likely mechanism of action of lidocaine is that the blockade of these voltage-gated sodium channels prevents the aberrant firing of irritable and hyper-excitable neurons, thereby decreasing neuropathic pain [5,27,37]. Lidocaine has proven to be at least mildly helpful in treating many neuropathic conditions, with some data showing it has been beneficial in PHN [26,38]. The most common side effects profile of topical lidocaine are site reactions and discomfort, while the more severe effects include cardiac arrhythmia and seizures. Relative contraindications include hypersensitivity to amide local anesthetics, affecting patients with heart conduction issues and those in overall poor health.
    - Capsaicin high-concentration patch
      - Capsaicin, the active ingredient in chili peppers, has been shown to have some mild analgesic properties when it comes to neuropathic pain. Its mechanism of action is on transient-receptor potential cation channel subfamily V member 1 (TRPV1) [27,30], which has its primary site of action on peripheral nociceptors, causing desensitization and dysfunction [5,30]. At the time of writing this article, the authors could find no long-term studies on the efficacy in long-term use. Common side effects are a burning sensation, site erythema, and, in severe cases, neurotoxicity [39].
    - Tramadol
      - Tramadol acts as both an μ receptor agonist and a serotonin-norepinephrine reuptake inhibitor. Researchers believe it works in combination with the modulation of descending adrenergic pathways and the peripheral μ activity, as well as exerting effects on μ receptor agonists at the spinal cord level, for which this drug has benefits. Despite many contradictory studies, there is little evidence that tramadol is effective at treating neuropathic pain. Side effects commonly seen include constipation, sedation, dizziness, and other GI disturbances [30]. The contraindications affect those who use MOA inhibitors, those with a history of GI disturbances, and those who are or may become suicidal.
  C Third-line treatments
    - Botulinum toxin A
      - There is limited evidence that the subcutaneous injection of botulinum toxin A has aided patients with some forms of focal neuropathies. The mechanism of this drug is not yet fully understood. However, researchers hypothesize it has neuromuscular actions in which the SNARE synaptobrevin complexes cannot release neurotransmitters, thereby helping with spontaneous neural discharges [40].
    - Opioids
      - There is weak evidence that opioids may be of some use regarding neuropathic pain. Acting peripherally and centrally on μ receptors, researchers hypothesize that opioids can aid the inhibition of central sensitization. However, when it comes to the efficacy of opioids in treating neuropathic pain or any other pain condition, one must consider the risks of the dependency, abuse, and diversion of these controlled substances [41].
    - Low Dose Naltrexone and Naloxone
      - Naltrexone and naloxone are traditionally considered to be opioid antagonists. However, at subclinical dosing, they are shown to reduce glial inflammation and even potentiate opioid analgesia. The reduction in the glial inflammatory response is thought to be due to regulating toll-like receptors 4 (TLR4) in connection with upregulating opioid signaling through transient blockade. With dosing intervals of less than 1 µg/day, naltrexone is thought to potentiate opioid analgesia through its actions on filamin A, a scaffolding protein known to be involved in sensory signaling. Although studies are limited regarding the use of opioid antagonists for the treatment of neuropathic pain, they have been shown to be beneficial for postoperative pain by reducing opioid usage and, thus, mitigating the opioid-related side effects. Low-dose naltrexone has shown benefit in the treatment of various conditions, including multiple sclerosis, chronic regional pain syndrome, fibromyalgia, and Crohn’s disease [42]. Although side effects have not been specifically studied in the treatment of neuropathic pain, there is evidence of the efficacy of the treatment of Crohn’s disease using low-dose naltrexone. A dose of 0.1 mg/kg, while not exceeding 4.5 mg, was shown to have a similar side effect profile to that of placebo, indicating limited toxicity when dosed appropriately in children.
2. Interventional treatments
  - Studies of interventional treatment regarding neuropathic pain are limited. Some data suggest that spinal cord stimulators can be an effective treatment option for neuropathic pain states, and they were recently approved by the FDA as a definitive treatment for painful diabetic neuropathy. Regarding the recently developed 10 kHz, paresthesia-free modality, it has been shown to be both efficacious and safe for treating neck, back, leg, and upper extremity pain. Studies have shown diminished pain scores following the 10 kHz SCS, showing improvements in function and quality of life, increased patient satisfaction, and the reduction in supplemental medication use [43]. The leading hypothesis for spinal cord stimulation is thought to be related to the gate control therapy mentioned earlier in this paper, as by overstimulating the sensory input to the spinal cord, the spinal cord stimulator impedes the noxious stimuli from reaching the regulatory centers in the spinal cord and brain stem. For completeness’ sake, the authors researched information on intrathecal pumps and deep brain stimulations, but at the time of writing this article, evidence is limited [41].

1. Pediatric Pain

Pediatric pain management is a growing field of pain management. Historically, pediatric primary care providers, pediatric subspecialists, and adult pain management providers have provided the bulk of pediatric subspecialty care to children with acute and chronic pain conditions due to the lack of subspecialty trained pediatric pain providers available for referrals. Over the last twenty years, there has been remarkable growth in and development of the subspecialty of pediatric pain management through greater access to training programs and investment in program development by many children’s hospitals across the globe. This has resulted in a greater understanding of the true incidence of chronic and, more specifically, neuropathic pain conditions in children and adolescents. Pediatric neuropathic pain is often underdiagnosed in part due to limited knowledge of the underlying mechanisms and limited exposure among non-pediatric-pain-trained providers. When providers without subspecialty training in pediatric pain management approach these patients, workup has been shown to be ineffective or lacking appropriate diagnostic criteria. For example, it has been shown that in children, there are more referrals to other providers prior to referral to the appropriate pain management centers. This overall delay in diagnosis and treatment leads to excessive, unnecessary workup, thus leading to socioeconomic burdens for patients and their families [44]. One of the most common issues that many providers come across in the field of pediatric pain management is the overall underdosing of the appropriate medications, even when the correct diagnosis is made [45]. Generally, this is due to the complex side effect profiles for many of the medications used to treat neuropathic pain, as well as the overall weight-based dosing guidelines set in place for younger children. There is, however, a bright side to the field of pediatric pain, as many children are able to recover meaningful function, and some (depending on the inciting factors) outgrow their symptoms in time with appropriate treatment and supportive care [44]. For the purpose of this review, the authors are unable to address the broader topic of pediatric pain management but will discuss several of the more common presentations and treatments available that have not previously been discussed in the adult-related sections.

- Genetic Pain Conditions

There are many genetic conditions that can predispose individuals to neuropathic pain states. Not all of them can be covered in this review; however, several more prevalent conditions are worth noting. Erythromelalgia, as mentioned previously, can also present in early childhood and cause significant distress for both pediatric patients and their caregivers. As a condition with some known and unknown mutations in sodium channel conductance, medications such as topical lidocaine can interact with and decrease the overall excitability of these nociceptive fibers. This has been shown to be partially effective at reducing the duration and severity of flares [5]. Other genetic conditions such as Fabry’s disease, which is an X-linked mutation affecting the *GLA* gene, which codes for α-galactosidase enzyme responsible for breaking down globotriaosylceramide that can build up in neuronal cells, leading to dysfunction and overall neuropathic pain states, as well as various other effects within the body, can also be present. Enzyme replacement has also been shown to ameliorate the systemic effects of the disease, but it has shown limited efficacy in terms of preventing the development of neuropathic pain. Of note, there are some data to support the notion that despite being unable to prevent the development of pain, there was a mild improvement in pain scores after the initiation of enzyme replacement therapy [46]. Firstly, second- and third-line medication options have been shown to be most effective, but in rare cases, interventional treatments can also be attempted to mediate the pain [44].

B. Anatomic compression and direct nerve injury

Similar to adults, the pediatric population is also subject to various insults to the peripheral and central nervous systems via direct injury to the associated nerves [44]. As many of these topics were discussed previously, they will only be briefly mentioned in this section. Some of the more common presentations of neuropathic pain as a result of direct injury include phantom limb pain as a result of amputation both surgical and congenital, brachial plexus avulsion as a result of trauma or difficult delivery at the time of birth, femoral neuropathy as a result of cardiac catheterization, or ilioinguinal neuralgia as a result of inguinal herniorrhaphy [46]. The mechanism associated with the development of these neuropathic states is consistent with similar injuries in the adult population. Often times, in the younger population, it can be difficult to assess the abnormal sensory disturbances due to age and their inability to appropriately describe the associated paresthesia/dysesthesias [44].

C. Complex Regional Pain Syndromes (CRPS I/CRPS II)

For the purposes of this review, CRPS is described as an individual topic, despite falling into both the idiopathic and direct nerve injury categories. CRPS is a poorly understood neuropathic pain state that falls into two categories: CRPS I, which was previously known as Reflex Sympathetic Dystrophy (typically seen when there is no apparent injury to the peripheral nerve), and CRPS II, previously known as “Causalgia”, in which there is an identifiable insult to the nervous system as a result of trauma, surgery, or disease process. Despite the inciting nature of CRPS, the symptoms of both type I and II are the same. There are typically three stages that the disease process could potentially take: acute, subacute, and chronic—all of which can lead to a resolution or progression to more permanent symptomatic presentations. While CRPS encompasses the typical paresthesia, allodynia, hyperalgesia, and hypersensitivities seen in typical neuropathic pain, it also presents with other features, such as skin color changes, skin temperature changes, swelling of the limb or joints in the affected area, abnormal sweating, muscle wasting, altered bone growth, and, lastly, hair and nail growth changes. The silver lining, however, is that in many cases of pediatric CRPS, the symptoms will diminish or even disappear altogether over time. Physical therapy, as well as first- and second-line medications, in combination with cognitive behavior therapy, have been shown to be efficacious in the treatment of CRPS [46].

D. Autoimmune Neuropathic Conditions

Although many of these conditions fall under the category of painful polyneuropathies, the authors felt it was worth mentioning several of the more common autoimmune causes of neuropathic pain, which can be important to keep in mind when coming up with differential diagnoses in the pediatric population [44]. Examples include Guillain-Barre syndrome, HIV/AIDs, Lupus, Rheumatoid arthritis, or even Sjogren’s syndrome, which should be considered when assessing a new pediatric patient for neuropathic pain; many of these illnesses have other more prominent associated features, leading to a definitive diagnosis. In many autoimmune/systemic inflammatory diseases, the first priority is to treat the underlying pathology, which, in many cases, aids pain relief. Used in combination with systemic therapy, medication, physical therapy, CBT, and interventional techniques have some limited data showing overall improvement in neuropathic pain [45].

E. Cognitive Behavioral Therapy (CBT)

In recent years, there has been a push to incorporate a more wholistic approach to addressing pain and the psychosocial effects that it can have, especially in the pediatric population [44]. There are some data that show that, in combination with the appropriate medication choices and physical therapy, CBT can provide an extra benefit in the treatment of pediatric pain. Early referral to an appropriate therapist can have a very positive impact not only on pain but also on the overall psychological outlook of both the patient and their immediate family [45].

## Data Availability

Not applicable.
